# Supplementary material for: Identification of photoperiod-induced specific miRNAs in the adrenal glands of Sunite sheep (Ovis aries)
Source: Front Vet Sci. 2022 Jul 22;9:888207. doi: 10.3389/fvets.2022.888207 (PMC9354845; doi:10.3389/fvets.2022.888207)
Supplement: Supplementary file 1 [file Data_Sheet_1.ZIP › Supplementary file/miRDeep2 file.zip!/miRDeep2 file/Novel_Structure/SP42A1/Novel_699.pdf]

Provisional ID	:	NC_019483.2_25955
Score total	:	12
Score for star read(s)	:	3.9
Score for read counts	:	4.2
Score for mfe	:	2.3
Score for randfold	:	1.6
Score for cons. seed	:	
Total read count	:	20
Mature read count	:	19
Loop read count	:	0
Star read count	:	1

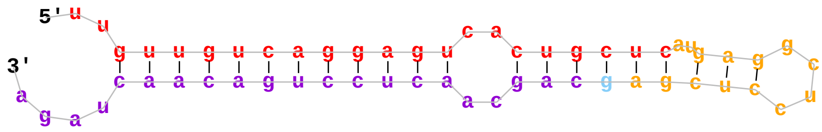

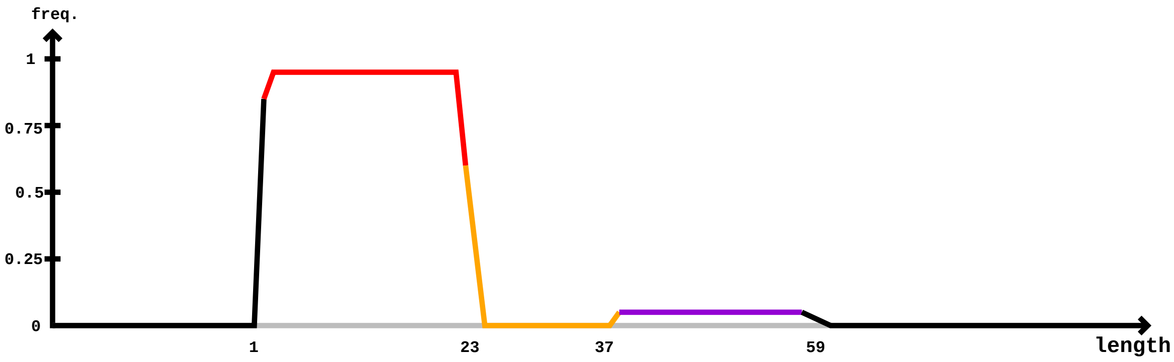

Mature Star

```
5'- gacgauguaggcucacccacuuguugucaggagucacugcucaugaggcuccucgagcagcaacuccugacaacuagaagggagggcagagcaucagcgcagccccugugga   -3'    obs
    gacgauguaggcucacccacuuguugucaggagucacugcucaugaggcuccucgagcagcaacuccugacaacuagaagggagggcagagcaucagcgcagccccugugga          exp
    ...(((((..((((.(((.((.((((((((((((..((((((..(((....)))))))))..)))))))))))).))..))).))))...)))))..(((((...)))))..   reads    mm    sample
    ....................uuguugucaggagucacugcu.......................................................................   7        0     seq
    ....................uuguugucaggagucacugcuc......................................................................   10       0     seq
    .....................uguugucaggagucacugcuc......................................................................   2        0     seq
    .........................................................cagcaacuccugacaacuaga..................................   1        0     seq
```
